# Comparison of the Effectiveness of Four Bariatric Surgery Procedures in Obese Patients with Type 2 Diabetes: A Retrospective Study

**DOI:** 10.1155/2014/638203

**Published:** 2014-05-22

**Authors:** Sylvie Pham, Antoine Gancel, Michel Scotte, Estelle Houivet, Emmanuel Huet, Hervé Lefebvre, Jean-Marc Kuhn, Gaetan Prevost

**Affiliations:** ^1^Department of Endocrinology, Diabetes and Metabolic Diseases, University Hospital of Rouen, 76031 Rouen, France; ^2^Department of Digestive Surgery, University Hospital of Rouen, 76031 Rouen, France; ^3^Department of Biostatistics, University Hospital of Rouen, Institut de Recherche et d'Innovation Biomédicale, Normandie University, 76031 Rouen, France; ^4^INSERM U982 Neuronal and Neuroendocrine Differentiation and Communication, Institut de Recherche et d'Innovation Biomédicale, Normandie University, University of Rouen, 76821 Mont Saint Aignan, France

## Abstract

*Aim*. The aim of the present retrospective study was to evaluate the efficacy of four bariatric surgical procedures to induce diabetes remission and lower cardiovascular risk factors in diabetic obese patients. Moreover, the influence of surgery on weight evolution in the diabetic population was compared with that observed in a nondiabetic matched population. *Methods*. Among 970 patients who were operated on in our center since 2001, 81 patients were identified as type 2 diabetes. Laparoscopic adjustable gastric banding (GB), intervention type Mason (MA), gastric bypass (RYGB), and sleeve gastrectomy (SG) were performed, respectively, in 25%, 17%, 28%, and 30% of this diabetic population. *Results*. The resolution rate of diabetes one year after surgery was significantly higher after SG than GB (62.5% versus 20%, *P* < 0.01), but not significantly different between SG and RYGB. In terms of LDL-cholesterol reduction, RYGB was equivalent to SG and superior to CGMA or GB. Considering the other cardiovascular risk factors, there was no significant difference according to surgical procedures. The weight loss was not statistically different between diabetic and nondiabetic matched patients regardless of the surgical procedures used. *Conclusion*. Our data confirm that the efficacy of surgery to treat diabetes is variable among the diverse procedures and SG might be an interesting option in this context.

## 1. Introduction

Bariatric surgery has proven to be a treatment of choice for morbid obesity [1, 2]. It is recommended for patients with body mass index (BMI) above 40 kg/m^2^ or higher than 35 kg/m^2^ when associated with comorbidities which include the different components of metabolic syndrome and type 2 diabetes [3, 4]. Weight loss obtained after bariatric surgery is associated with a highly significant reduction in cardiovascular risk factors [5–7]. More recently, improvement or remission of diabetes has been observed following bariatric surgery in obese patients with type 2 diabetes and mortality rate linked to diabetes was consequently significantly reduced [1, 2, 8–12]. Weight loss induced by bariatric surgery is a major factor of diabetes improvement [11, 13]. However, in several studies, the resolution of diabetes has often been observed before a significant weight loss has been obtained [14–17]. The early postsurgical improvement of diabetes suggested a major physiopathological role for changes in gut hormone secretion [18]. As a matter of fact, a decrease in plasma levels of ghrelin, an orexigenic peptide, has been described following gastric bypass for morbid obesity [19–22]. The involvement of other intestinal peptides like GLP-1 (hindgut hypothesis) [23–26] and neuropeptide YY [22, 27–30] or a decreased secretion of anti-incretin hormones (foregut hypothesis) [31, 32] has been proposed to explain the rapid remission of diabetes after bariatric surgery. Otherwise, Roux-en-Y bypass (RYGB) that excludes the duodenum from the nutriments' route and profoundly modifies the gut microbial metabolic cross-talk [33] has been shown to improve insulin resistance more rapidly than sleeve gastrectomy [15].

These observations raised the question of the choice of chirurgical procedure for treating diabetes. Most studies have compared two by two procedures. Using this approach, RYGB and sleeve gastrectomy (SG) seem to be more efficient for treating diabetes than gastric banding (GB). However, these studies failed to provide clear conclusions owing to the great heterogeneity of the results. For instance, depending on the mode of surgery used (restrictive, malabsorptive, or combined) and study design, the diabetes remission rate varied from 45 to 97% of patients [5, 9, 12, 34–39]. In our center, four surgical procedures have been used for the last 15 years to treat obesity, GB, SG, calibrated gastrectomy type Mason (CGMa), and RYGB. The aim of the present retrospective study was to evaluate the efficacy of these four procedures to induce diabetes remission and lower cardiovascular risk factors. Moreover, the influence of surgery on weight evolution in the diabetic population was compared with that observed in a nondiabetic population matched for sex, age, BMI, and surgical procedure.

## 2. Patients and Methods

We have conducted a retrospective case-control comparative study aimed at evaluating the impact of bariatric surgery on type 2 diabetes. Among 970 patients who underwent bariatric surgery in our center since 2001, 81 patients were identified as type 2 diabetic (ICD coding). All patients had undergone a medical supervised therapy for weight loss, for at least 12 months before bariatric surgery.

Preoperatively, they were evaluated for medical or surgical history, and treatment used was recorded. A clinical examination was performed and risk factors and comorbidities, including arterial hypertension and sleep apnoea syndrome, were collected. The serum levels of biological markers (fasting glycemia, glycosylated hemoglobin, total cholesterol, HDL- and LDL-cholesterol, and triglycerides) were measured.

Then, one of the four following surgical procedures was used: GB (20 patients), CGMa (14 patients), SG (24 patients), and RYGB (23 patients).

After surgery, the weight change could be followed for 24 months and biological parameters for 12 months. 23% of the patients were lost for follow-up.

The weight changes in patients with diabetes were also compared to those observed in obese patients without diabetes and matched for age, sex, BMI, and type of surgery.

## 3. Statistical Analysis

The evolution of diabetes has been chosen as the primary end point of the study. It was evaluated on the basis of a score of effectiveness. Remission of diabetes (score 1) was defined as HbA1c level lower than 6.5% and interruption of antidiabetic treatment. Improvement (score 2) was defined as either a decrease in HbA1c level or a reduction in the antidiabetic treatment. Score 3 corresponded to a stabilisation of diabetes and score 4 to a degradation of the glycaemic pattern. As the latter is a qualitative parameter, statistical analysis of the data used a *χ* ^2^ test. Similar scores and statistical procedures were used to evaluate the changes in the following comorbidities: arterial hypertension, sleep apnoea syndrome, and serum lipid pattern.

The serum HbA1c levels, measured, respectively, before and 6 to 12 months after surgery, were compared with the Wilcoxon test. The nonparametric test of Kruskal Wallis was used to evaluate this biological parameter as a function of the type of surgery employed.

The weight changes were initially analysed using ANOVA for two factors: time and type of surgery. Depending on the results of this first analysis, a second statistical evaluation using an ANOVA for one factor (type of surgery) was performed. Complementary tests of comparison two by two (Newman and Keuls) were done for the different time points where ANOVA (one factor) was statistically significant.

Statistical analyses were performed with Statview software (5th version, SAS Institute, North Carolina). Statistical significance was considered for a *α* risk of 5%.

## 4. Results

The main characteristics of diabetic patients are summarized in Table 1. The mean diabetes duration was 8 ± 8 years. According to WHO criteria, 67 patients (82.7%) harboured a profile of metabolic syndrome. The number of patients with cardiovascular risk factors was similar whatever the type of surgery performed. 38% of the patients suffered from micro- and/or macrovascular complications of diabetes and 24 (29.6%) complained of signs of depression. 10 patients were treated by diet alone, 44 patients (54%) by oral antidiabetic drugs (OAD), 7% by insulin, and 25% by OAD plus insulin.

**Figure 1:**
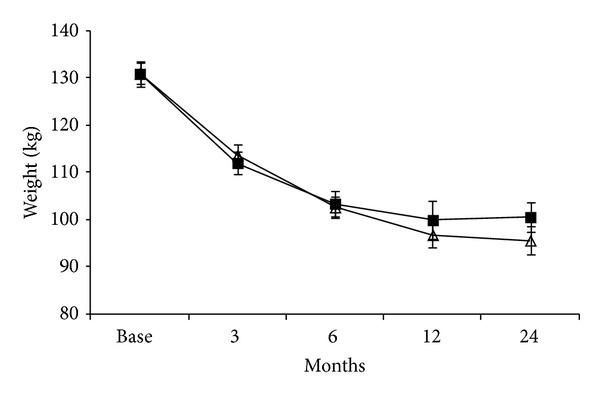
Overall changes in weight (mean ± SEM) in patients treated with bariatric surgery. (Δ) obese patients without diabetes; (■) obese patients with type 2 diabetes.

**Figure 2:**
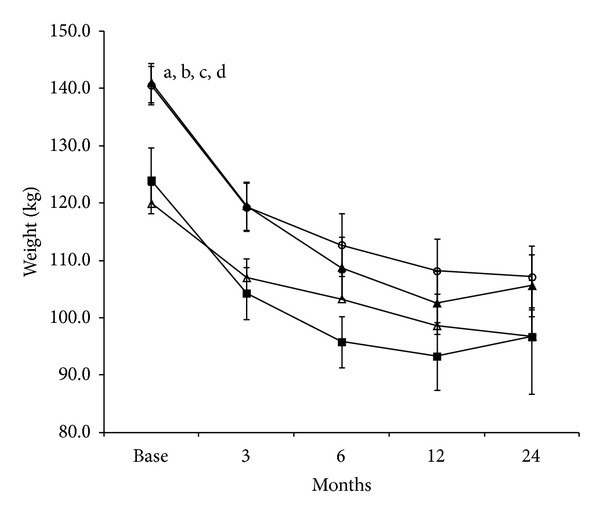
Changes in weight (mean ± SEM) induced by the different types of bariatric surgery procedures in obese diabetic patients. (Δ) gastric banding (GB); (*ο*) calibrated gastrectomy type Mason (CGMa); (▲) sleeve gastrectomy (SG); (■) Roux-en-Y gastric bypass (RYGB). ^a^ *P* < 0.05 CGMa versus GB; ^b^ *P* < 0.05 SG versus GB, ^c^ *P* < 0.05 CGMa versus RYGB, ^d^ *P* < 0.05 SG versus RYGB.

Bariatric surgery induced a significant (*P* < 0.001) weight loss in both groups of obese patients (Figure 1). One year after surgery, weight was reduced by 30.7 ± 2.1 and 37.2 ± 2.6 kg in patients with and without diabetes, respectively (Figure 1). Basal weight before surgery was significantly different according to the surgical procedure (*P* = 0.002) (Figure 2) so further ANOVA analysis has been performed on the weight difference. A significant weight loss (*P* < 0.01) was obtained after each surgical procedure, reaching 26.0 ± 2.0% (−6.7 ± 4.6 kg, GB), 28.0 ± 1.6% (−27.8 ± 5.4 kg, CGMa), 41.0 ± 3.3% (−32.2 ± 5.0 kg, SG), and 43.0 ± 2.9% (−28.2 ± 4.5 kg, RYGB) of the initial weight on month six after surgery. Repeated measure ANOVA analysis (time × surgical procedure) revealed significant differences between the weight loss and the surgery procedure in the diabetic group (*P* = 0.007). The weight loss in the GB group compared to the three other procedures was statistically lower at 3, 6, and 12 months after the surgery.

**Figure 3:**
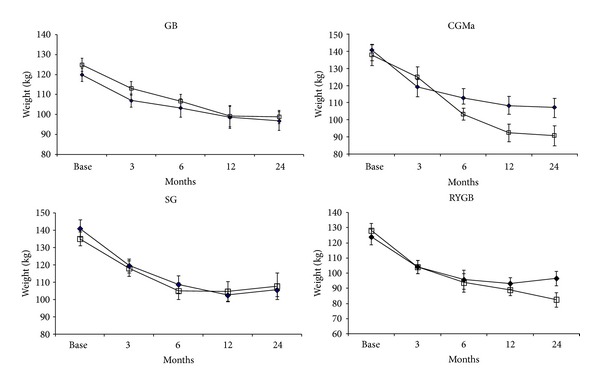
Changes in weight (mean ± SEM) induced by the four different types of bariatric surgery in nondiabetic and diabetic obese patients. Gastric banding (GB), calibrated gastrectomy type Mason (CGMa), sleeve gastrectomy (SG), and Roux-en-Y gastric gypass (RYGB). (□) Obese patients without diabetes; (■) obese patients with type 2 diabetes.

The weight loss was not statistically different between diabetic and nondiabetic matched patients, regardless of the surgical procedures performed (Figure 3).

**Figure 4:**
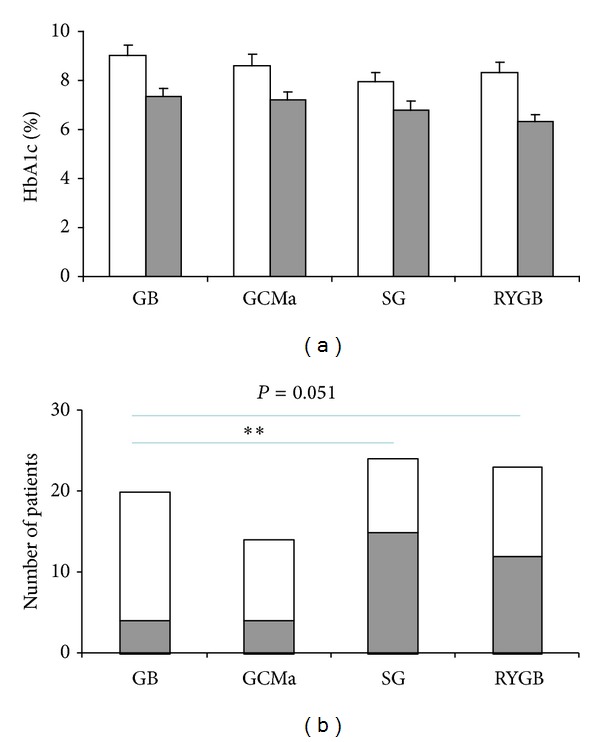
Impact of bariatric surgery on the evolution of diabetes in diabetic obese patients. (a) changes in serum HbA1c levels (mean ± SEM) observed in obese diabetic patients following each of the four different types of bariatric surgery. White columns represent serum HbA1c levels measured before surgery and grey toned columns represent the value measured one year after surgery. (b) Number of patients in whom remission of the diabetes was observed after bariatric surgery. White columns represent the total number of patients operated for each type of surgery. Grey toned zones show the number of patients in whom a remission of diabetes was observed. Gastric banding (GB), calibrated gastrectomy type Mason (CGMa), sleeve gastrectomy (SG), and Roux-en-Y gastric bypass (RYGB). **P < 0.01.

In the whole group of obese patients with diabetes, a statistically significant decrease in serum HbA1c level from 8.4 ± 0.2% to 6.8 ± 0.16% (*P* < 0.001) was observed one year following surgery. As a function of type of surgery, serum HbA1c level dropped from 9.01 ± 0.44 to 7.33 ± 0.33% (GB), 8.61 ± 0.47 to 7.21 ± 0.32% (CGMa), 7.94 ± 0.38 to 6.81 ± 0.33 (SG), and 8.31 ± 0.43 to 6.34 ± 0.27 (RYGB, *P* < 0.001) (Figure 4). In terms of decrease in HbA1c levels, no statistical difference was found between the different surgical procedures.

Considering the overall surgical approach, diabetes significantly (*P* = 0.015) improved after surgery. A remission was observed in, respectively, 20% of patients after GB, 29% after CGMa, 62.5% after SG, and 52% after RYGB. Diabetes remission was clearly better after SG than following GB (*P* = 0.0026). Conversely, the superiority of RYGB on GB was only at the limit of statistical significance (*P* = 0.051) (Figure 4).

Arterial hypertension resolved in 12% of the patients and was improved in additional 7% of them after bariatric surgery. However, no statistically significant difference in either arterial blood pressure or antihypertensive drug need was observed between the four surgical procedures. The intensity of sleep apnoea syndrome and the need of continuous positive airway pressure were not modified, on a statistical basis, after each mode of surgery despite the weight loss. In contrast, in the group of diabetic patients considered as a whole, sleep apnoea syndrome disappeared or was improved after bariatric surgery in, respectively, 20% and 90% of cases. The serum levels of HDL-cholesterol did not significantly change during the postsurgery follow-up. In contrast, serum triglycerides fell in 76% of the patients but the comparison between the four different modes of surgery did not reveal any significant difference among them. Serum LDL-cholesterol level was significantly (*P* = 0.0034) reduced after surgery. The decrease in LDL-cholesterol was significantly (*P* = 0.01) higher after CGMa than following GB and significantly higher (*P* = 0.03) after RYGB than following CGMa. RYGB and SG induced similar decreases in serum LDL-cholesterol level.

## 5. Discussion

Bariatric surgery has been proven to be an effective approach for the treatment of morbid obesity in adults with BMI > 40 kg/m^2^ [2, 5, 17]. An increasing body of evidence also emphasizes the bariatric surgery benefit for the treatment of the type 2 diabetes in obese patients. Moreover, the new therapeutic algorithms of type 2 diabetes suggest an earlier surgical intervention for increasing the likelihood of remission of diabetes [4, 40]. However, the question of the choice of surgical procedure to be used in this purpose remains yet largely debated and many variations in selection of the surgical technique are observed among obesity care centres. Because randomized studies are scarce and very difficult to realize, until now most statements are issued from observational studies and meta-analyses. In this context, the choice for the surgical procedure remains open according to IDF recommendations [4].

Our study is the first one comparing four surgical procedures of bariatric surgery on the remission rate of diabetes, weight loss, and reduction of comorbidities in obese patients with type 2 diabetes with similar effectiveness in the diverse groups of patients. Although weight loss was similar following each type of bariatric surgery, significant differences were observed in the remission rate of type 2 diabetes depending on the surgical method used. SG or RYBG gave no different results but were followed by better remission rates than after GB. The lack of standardization for the criteria of remission of diabetes between studies introduces a limit to the strength of the comparison with those previously published on this topic. However, our results agree with those of Abbatini et al. [37] and Campos et al. [40] who showed a superiority of RYGB on GB on the remission rate of type 2 diabetes and with the conclusions of the meta-analysis by Buchwald et al. [11]. It has been proposed that differences in the secretion of gut hormones (ghrelin, GLP-1, peptide YY) occurring after SG or RYGB may play a pivotal role in diabetes regression by acting on appetite [41, 42], improving insulin sensitivity, and restoring the first phase of insulin secretion [23, 27, 28], all effects which, concomitantly to weight loss, participate to the improvement of diabetes.

In our study, the best rates of remission of diabetes were observed after performing either SG (62.5%) or RYGB (52%). They seem to be quite lower than those previously reported but the results are variable from one study to the other. For instance, Schauer et al. [43] described a resolution of diabetes in 83% of the 240 patients operated by laparoscopic RYGB and followed for a five-year period. However, a recent randomised trial reported 42% remission after RYGB and 37% after SG 12 months after the surgery, with no difference between the 2 surgical groups in diabetic obese patients [44]. This discrepancy of the efficacy could be explained by the lack of standardisation for remission criteria as well as phenotypic differences among patients. Indeed, our patients were quite older and one-third exhibited diabetic complications, suggesting a more pronounced diabetic state.

No significant difference in weight changes was observed in the group of nondiabetic obese patients by comparison with diabetic patients regardless of the type of surgery performed. By comparison with nondiabetic patients, a lower decrease in weight has been previously observed in obese diabetic patients treated with RYGB [45] in relation to the modalities of the treatment for diabetes. In this study, patients requiring a more powerful treatment of diabetes lost less weight. Conversely, in the present study, the proportion of patients who had the diverse modalities of treatment, that is, diet alone, oral hypoglycemic drugs, and/or insulin, was similarly distributed between the four surgical procedures; a direct incidence of the therapy for diabetes on the pattern of weight loss appears unlikely.

As frequently observed in retrospective studies, the preoperative characteristics of the patients were significantly different between the groups. This can be explained by the fact that the mode of surgery had been chosen according to the patient's profile. In fact, while GB or RYGB were performed in patients with lower BMI (40–45 kg/m^2^) and in those suffering from nibbling, SG was preferentially proposed in patients with BMI > 50 kg/m^2^ or with significant comorbidities.

We observed a postsurgical improvement in arterial hypertension and sleep apnoea syndrome in the group of diabetic patients considered as a whole. The higher number of comorbidities found in our severely obese patients, whose diabetes was imperfectly controlled as evidenced by high basal HbA1c levels, the long-lasting preoperative duration of diabetes which was frequently complicated (38% of cases), could explain the less pronounced improvement than that reported in other studies [11]. Considering each of the four surgical procedures, none appears to be significantly more effective than others to improve arterial hypertension or sleep apnoea syndrome. Bariatric surgery was followed by a significant drop in serum triglycerides in agreement with previously published studies [6, 15, 46]. However, in contrast to reports of higher effectiveness of RYGB to reduce serum triglyceride levels [15, 46, 47], we found no difference between the four types of surgery used. This observation likely results from the limited sizes of the patients groups. Similarly, no significant change was noticed in HDL-cholesterol. In contrast, RYGB appears as potent as SG to decrease LDL-cholesterol level, both being more effective than CGMa.

Like in the majority of retrospective investigations, the main limitation of our study is the absence of randomisation. However, randomised trials are particularly difficult to realize in this field and even not conceivable for four different surgical procedures. Another classical limitation in this type of study is the high percentage of patients lost to follow-up. However, it is important to notice that 30% of these patients declared that they had stopped the specialized follow-up because of diabetes regression. This observation illustrates the difficulties to follow these patients and emphasizes the need for clinical investigators to develop collaboration with general practitioners.

To conclude, the results of our study suggest that among the four surgical procedures available for the management of obese patients with type 2 diabetes, SG and RYGB might be more efficient than CGMa and GB to improve DT2 one year after the intervention. SG seems to be in our study at least as efficient as RYGB to treat diabetes and other comorbidities. This observation, which needs to be confirmed in larger populations, is interesting because SG offers several other advantages. Especially, SG, which is a technically simpler method of surgery than RYGB, induces less vitamin deficiency and can be subsequently converted into RYGB if its effects are considered unsatisfactory. However, its greater safety and feasibility have not been clearly demonstrated [48].

## Figures and Tables

**Table 1:** Baseline characteristics of the obese patients with type 2 diabetes.

	GB	CGMa	SG	RYGB
Number of patients	20	14	24	23
Mean age (years)	44	46	48	45
Mean BMI (kg/m^2^)	46	52	52	46
Arterial hypertension, *N* (%)	14 (70)	10 (71)	18 (75)	19 (83)
Hypertriglyceridemia, *N* (%)	13 (65)	7 (50)	10 (42)	13 (57)
Hypercholesterolemia, *N* (%)	14 (70)	10 (71)	14 (58)	10 (43)
Metabolic syndrome, *N* (%)	17 (85)	10 (71)	22 (92)	18 (78)
Sleep apnoea syndrome, *N* (%)	6 (30)	10 (71)	14 (58)	10 (43)
